# Endoscopic Transnasal Transclival Removal of a Giant Hemangiopericytoma Involving the Clivus and C1-C2 Vertebrae: A Clinical Case and Literature Review

**DOI:** 10.7759/cureus.79040

**Published:** 2025-02-15

**Authors:** Alexey N Shkarubo, Diana S Adueva, Dmitry N Andreev, Suzanne A Galstyan, Ilya V Chernov, Andrey A Panteleyev

**Affiliations:** 1 Neurooncology, Federal State Autonomous Institution, N.N. Burdenko National Medical Research Center of Neurosurgery, Ministry of Health of the Russian Federation, Moscow, RUS; 2 Medicine, Russian Medical Academy of Continuous Professional Education, Moscow, RUS; 3 Pathology, Federal State Autonomous Institution, N.N. Burdenko National Medical Research Center of Neurosurgery, Ministry of Health of the Russian Federation, Moscow, RUS; 4 Orthopedics and Traumatology, Russian Children’s Clinical Hospital, Moscow, RUS

**Keywords:** clivus, craniovertebral junction, endoscopic transnasal transclival neurosurgery, hemangiopericytoma, skull base, solitary fibrous tumor

## Abstract

Hemangiopericytomas, or solitary fibrous tumors (SFT), are neoplasms of mesenchymal origin that are quite rare in oncological practice. These tumors often grow to large sizes before the onset of clinical symptoms, and in imaging studies, they closely resemble meningiomas, making diagnosis challenging. When treating intracranial hemangiopericytoma, it is necessary to ensure the most radical removal followed by radiation therapy. We present a rare case of transnasal transclival subtotal removal of a giant hemangiopericytoma/SFT of the craniovertebral junction and the C1-C2 vertebral level, not previously described in the literature. Based on the results of the literature review, it was determined that an accurate diagnosis during the instrumental and morphological examination is crucial for the future outcome of a patient with intracranial hemangiopericytoma, as confirmed in the presented case.

## Introduction

Hemangiopericytomas are aggressive tumors associated with a high frequency of recurrence and distant metastasis [1,2]. These tumors can reach significant dimensions without obvious clinical manifestations [3-5]. Primary clinical symptoms are caused mainly by the mass effect and vary depending on its localization - often manifested by headache, seizures, and focal neurological symptoms [6]. Hemangiopericytomas share similar clinical manifestations and radiological findings with meningiomas [7,8], which makes it difficult to differentiate these tumors.

In 2013, the World Health Organization's (WHO) classification of tumors of the central nervous system (CNS) combined hemangiopericytomas and solitary fibrous tumors (SFTs) into a single category. Continued discussions and analysis have revealed that hemangiopericytomas have not only similar histological characteristics with SFTs but are also linked with similar genetic abnormalities. Both tumors are of mesenchymal origin and are quite rare (0.1% of the total number of oncology patients). These tumors affect the skin and subcutaneous fat tissue (34.5%), skeletal muscles of the lower extremities (24.5%), retroperitoneal space (24%), and the head and neck region (17%) [9,10]. Intracranial SFTs are rare and account for 0.4% of all primary brain tumors [7,11,12].

Currently, there are no clinical observations of hemangiopericytomas of the clivus and craniovertebral junction in the literature. We present a rare clinical case of endoscopic transnasal removal of a giant hemangiopericytoma at the level of the clivus and C1-C2 vertebrae with a detailed description of imaging studies, morphological and immunohistochemical findings, and a review of available literature.

## Case presentation

A 37-year-old female patient underwent her first magnetic resonance imaging (MRI) of the head and neck in 2019 due to complaints of headaches and neck pain. The scan revealed a small tumor at the craniovertebral junction, specifically in the C1 vertebra region. In 2019, partial tumor removal was performed at a neurosurgical facility in the patient's area of residence. A laminectomy of the C1 and C2 vertebrae was conducted, with the histological diagnosis of anaplastic neurofibroma. Several months later, the patient underwent radiation therapy with a total radiation dose (TRD) of 54 Gy.

**Figure 1:**
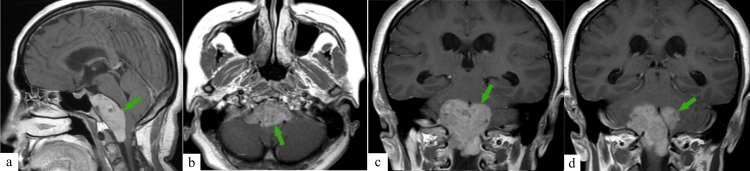
MRI of the brain before endoscopic transnasal transclival tumor removal. T1-weighted images with contrast enhancement (the tumor is indicated by the green arrows): (a) sagittal plane; (b) axial plane; (c and d) coronal plane. Tumor dimensions 61 mm x 50 mm x 32 mm (length x width coronal x width sagittal). MRI, magnetic resonance imaging

About four years after surgery, the patient experienced the onset of severe neck pain. An MRI revealed a small tumor in the region of the middle and lower clivus, extending to the level of the C1 segment. During the primary examination at our center, images taken seven months after the onset of neck pain were provided, showing a large tumor measuring 61 mm x 50 mm x 32 mm in the middle and lower clivus area, extending to the level of the C1-C2 segment of the cervical region (Figure 1). The tumor was complicated by compression of the pons, medulla oblongata, and the upper region of the cervical spinal cord. Signs of rapid growth of the tumor were noted over several months.

Available MRI sequences revealed a tumor mass with a heterogeneously hyperintense T2-weighted signal and an iso/hypointense T1-weighted signal. Periventricularly the T2-weighted white matter signal was increased. In diffusion-weighted imaging (DWI) mode, no pathological changes in the magnetic resonance (MR) signal were detected intracranially. However, after contrast administration, heterogeneous contrast enhancement was observed in the tumor.

**Figure 2:**
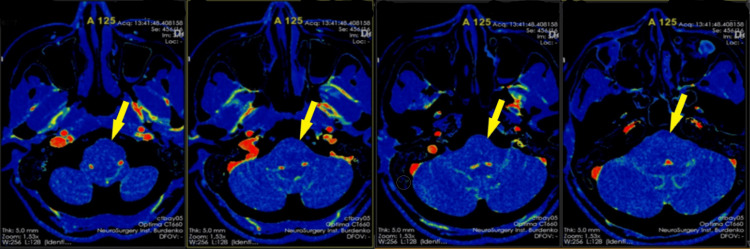
CT perfusion of the brain before endoscopic transnasal transclival tumor removal. CT axial plane images: tumor mass with low blood flow rates (indicated by the yellow arrow). CT, computed tomography

CT perfusion imaging revealed low blood flow rates, including cerebral blood flow (CBF) and cerebral blood volume (CBV): CBF = 29.17 mL/100 g/minute (reference: 24-34 mL/100 g/minute), CBV = 2.213 mL/100 g (reference: 1.182 mL/100 g). Additionally, mean transit time (MTT) was 11.74 seconds (reference: 1.019 seconds), and permeability surface area product (PS) was 6.903 mL/100 g/minute (reference: 3.970 mL/100 g/minute) (Figure 2).

**Figure 3:**
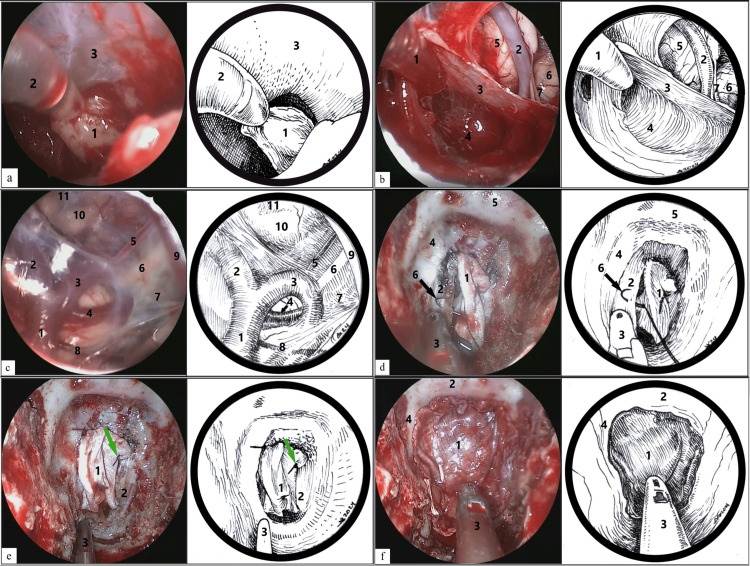
Intraoperative images of tumor removal and skull base defect reconstruction. Stages of the surgery: (a) Removal of soft grayish-yellow tumor tissue (1, tumor; 2, suction tip; 3, arachnoid membrane). (b) Post-resection view of the tumor bed in the caudal direction. There are no obvious tumor remnants (1, suction tip; 2, right vertebral artery; 3, arachnoid membrane; 4, the entire tumor within the visible area has been removed, and no obvious residual parts of the tumor are visible; 5, pyramid of the medulla oblongata; 6, olive; 7, XII n.). (c) Post-resection view of the tumor bed in the rostral direction (1, basilar artery; 2, right posterior cerebral artery; 3, left posterior cerebral artery; 4, right superior cerebellar artery; 5, right posterior communicating artery; 6, III n.; 7, arachnoid membrane; 8, pontine artery; 9, middle cerebral artery; 10, mammillary bodies; 11, Liliequist membrane). (d) Closure of the defect (1, fascia taken from the lateral surface of the patient’s left thigh; 2, dura mater [DMA]; 3, Blakesley Rhinoscopic Nasal Forceps; 4, clivus; 5, sella turcica; 6, a needle with 4/0 suture). (e) The green arrow indicates the suture placed on the dura mater and fascia (1, fascia; 2, dura mater; 3, Blakesley Rhinoscopic Nasal Forceps). (f) Second layer of fascia is placed over the reconstruction site and fixed with a Foley catheter to the base of the skull (1, second layer of fascia; 2, sella turcica; 3, Blakesley Rhinoscopic Nasal Forceps; 4, projection of the right carotid artery). Image credits: Dmitry N. Andreev.

An endoscopic transnasal transclival tumor removal was performed at our institution, followed by reconstruction of the dural and skull base defects using autologous tissues (Figure 3). Endoscopic access to the clivus area was carried out through staged trepanation. The basal dura mater was opened. The tumor was soft, moderately vascularized, and grayish-yellow in color. Removal was performed mainly by suction and rongeurs. The tumor tissue was anatomically separated from the adjacent brain structures by a firm arachnoid membrane. The tumor was completely removed in stages within the visible margins using 0°, 30°, and 45° endoscopes sequentially.

**Figure 4:**
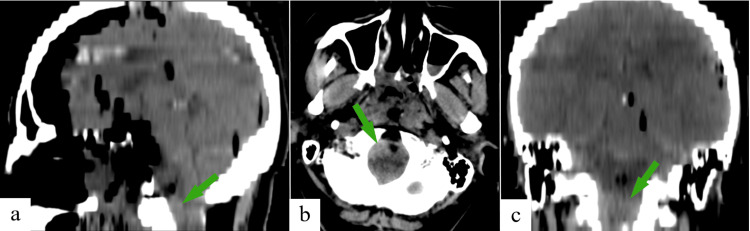
CT scan of the brain immediately after surgery. Postoperative pneumocephalus, subtotal tumor removal: (a) sagittal plane; (b) axial plane; (c) coronal plane (the arrows indicate the residual tumor fragment at the level of C1-C2). CT, computed tomography

Immediate postoperative CT images demonstrated insignificant pneumocephalus, a small residual tumor remnant, and no postoperative complications (Figure 4).

**Figure 5:**
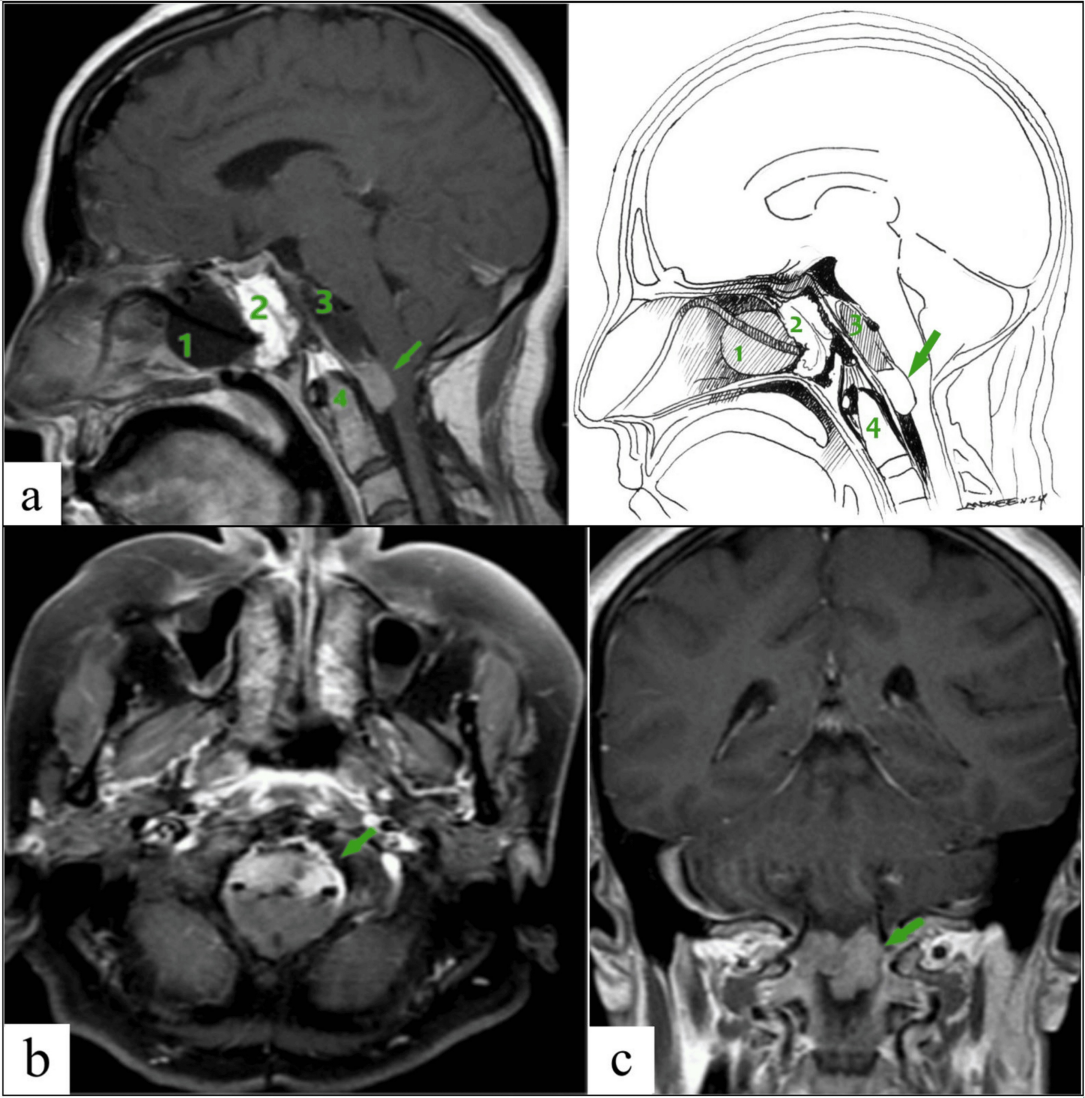
T1-weighted MR images of the brain on the fifth day after surgery with contrast enhancement (subtotal tumor removal). (a) Sagittal plane with illustration; (b) axial plane; (c) coronal plane (1, Foley catheter; 2, reconstruction of the skull base defect; 3, bed of the removed tumor; 4, odontoid process of the C2 vertebra, arrows indicate the residual fragment at the level of C1-C2). Image credits: Dmitry N. Andreev. MR, magnetic resonance

A follow-up MRI performed one month after the operation demonstrated a residual tumor fragment with homogeneous contrast enhancement, extending to the odontoid process of C2. Additionally, a complete regression of pneumocephalus was observed (Figure 5). The residual fragment was approximately 3% of the original tumor in volume. According to the extent of the tumor resection scale proposed by Frank and Pasquini [13], subtotal removal of the tumor was accomplished.

**Figure 6:**
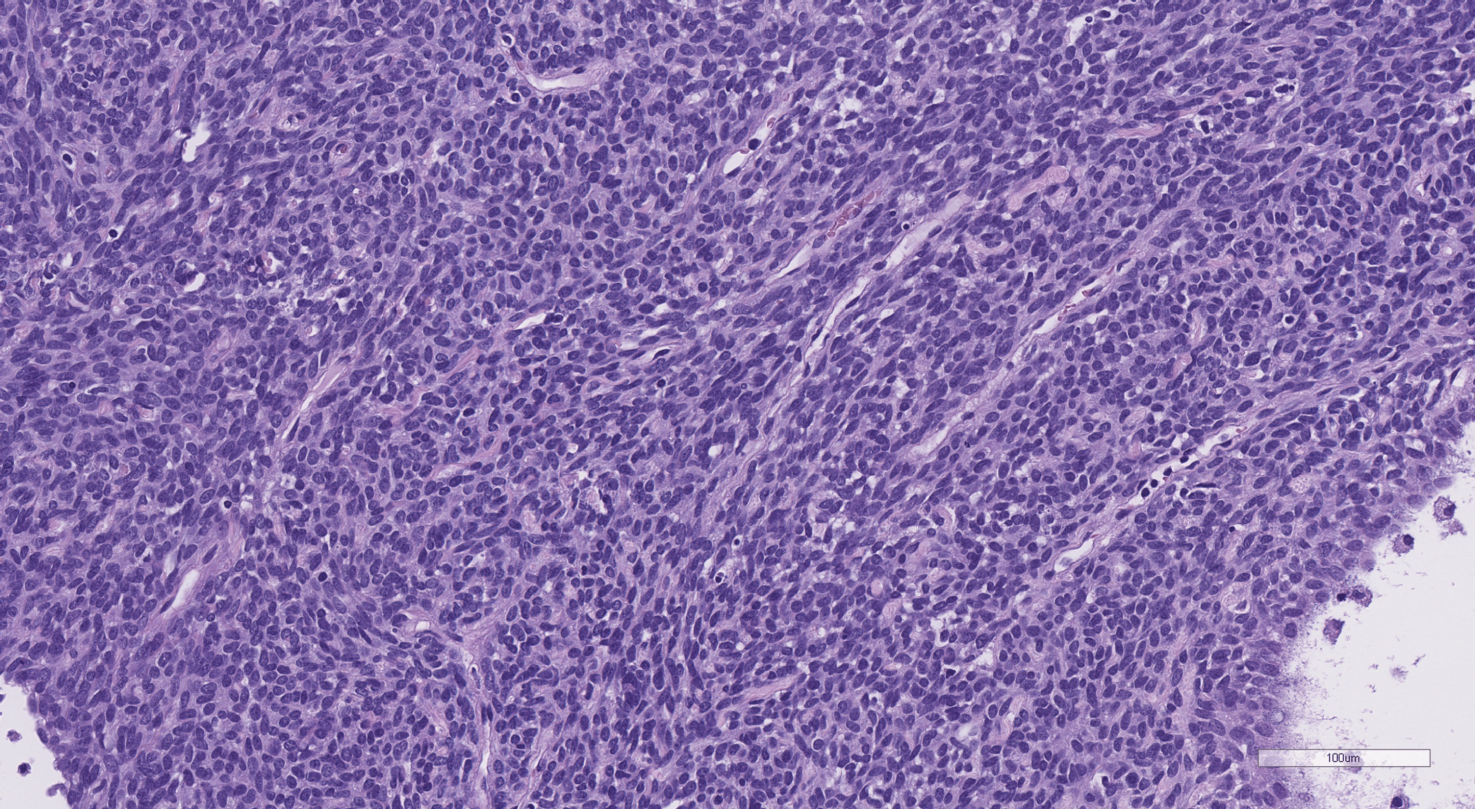
Histological preparation stained with hematoxylin and eosin (magnification x200).

The tumor tissue samples were fixed with 10% buffered formaldehyde, prepared according to the standard protocol, and embedded in a paraffin block. Histological sections were made from the paraffin block and stained with hematoxylin and eosin (Figure 6).

Histopathological analysis of the preparations demonstrated solid tumor fragments with a typical hemangiopericytic pattern. The cells were distinguished by the absence of cellular boundaries, scanty cytoplasm, and an increased nuclear-cytoplasmic ratio. The cell nuclei were moderately polymorphic, medium-sized, and predominantly ovoid in shape. The chromatin was normochromic, coarsely and finely dispersed, with 1-3 small, basophilic nucleoli. Mitotic activity was increased to six mitotic figures per 10 consecutive fields of vision at x200 magnification. Cell density was high. The tumor was richly supplied with thin, slit-like vessels, in places barely distinguishable at the light-optical level. Cystic cavities of various calibers were also noted. No foci of necrosis were detected within the examined material.

Immunohistochemical analysis demonstrated focal expression of CD34 and diffuse nuclear expression of STAT6, which is a pathognomonic immunophenotype for SFTs.

Based on histopathomorphological findings and immunophenotyping, the diagnosis was an SFT, CNS WHO grade II, per the 5th edition of the WHO Classification of CNS Tumors (2021).

By the time of discharge, the patient's neck pain had completely resolved. She was discharged on the 29th day in satisfactory condition with recommendations for further treatment, including radiation therapy up to a total dose of 54 Gy, scheduled two months after surgery.

## Discussion

Hemangiopericytomas are neoplasms originating from perivascular cells, developing mainly in soft tissues. They were first described in 1942 by American pathologists Arthur Purdy Stout and Margaret Ransone Murray [14,15]. Because of the difficulty of histologic differentiation, the tumor underwent several nomenclature reclassifications over the years, such as benign mesothelioma, solitary fibrous mesothelioma, and localized fibrous tumor [16]. Histologic examination of hemangiopericytomas demonstrates cells of variable shape, ranging from oval to spindle-shaped cells with minimal nuclear atypia and mitotic activity, that are arranged haphazardly or in short, ill-defined tufts. An extensive, branching vasculature and alternating hypercellular and hypocellular sclerotic foci are characteristic of these tumors [17,18]. Because of the nonspecific histologic appearance, immunohistochemical staining plays an important role in the diagnosis of SFTs. SFTs are immunoreactive for CD34 and STAT6 and are variably positive for CD99, but negative for muscle, epithelial, and neural markers [19]. It is important to note that CD34 is not specific enough for a definitive SFT diagnosis. Strong nuclear staining for STAT6 has been used to reliably differentiate SFTs from other soft tissue tumors [20]. The presence of focal CD34 expression and diffuse nuclear STAT6 expression supported the diagnosis of hemangiopericytoma in our clinical case.

Hemangiopericytomas cannot be differentiated from the much more common meningiomas based on MRI data alone [8].

These tumors are predominantly found in patients in their thirties and forties, but up to 10% of cases are diagnosed in children [6]. Published cases demonstrate no relationship between the development of hemangiopericytomas and the patient's gender.

To date, surgical resection remains the method of choice for the treatment of these neoplasms [5,21,22]. The degree of malignancy of these tumors can vary from G1 to G3, which determines their growth rate [11]. The frequency of *local* recurrence is relatively high, even after radical removal of SFTs; some authors reported rates as high as 50%. Postoperative radiation therapy, however, substantially reduces the relapse frequency [12,23-30]. In cases of hard-to-access tumor localization, partial tumor resection in combination with postoperative radiation therapy is preferable [1,30,31]. According to most authors, the most effective TRD was 50-60 Gy [12,24]. It should be noted that, according to several studies describing extensive surgical resection of these tumors, accompanied by a low risk of postoperative complications and low mortality [32,33], midline skull base tumors were predominantly resected using the endoscopic transnasal transclival (posterior extended) approach [34,35].

According to Galanis et al., only one out of seven patients experienced tumor regression when undergoing chemotherapy with doxorubicin [2]. However, some authors report effective use of a treatment protocol based on a combination of ifosfamide and epirubicin. Mena et al., who observed 94 cases of hemangiopericytomas, reported a 70% recurrence rate and metastases in 27% of cases. Up to 20% of hemangiopericytomas can metastasize distantly - usually to the liver, lungs, or skeletal bones [6,7,11], and less frequent cases of metastasis to the kidneys [36,37]. According to available literature, metastases were observed on average 63 to 99 months after diagnosis. A case of hemangiopericytoma metastasis 20 years after diagnosis was also described. In most cases, tumor recurrence is associated with the presence of a distant metastasis [38].

Postoperative radiotherapy reduces the risk of local recurrence, but not the development of metastases in the central nervous system and other localizations. It also does not exclude the risk of recurrence [39]. The largest size of tumors recommended for radiosurgery was reported to be 25 mm [40]. Radiosurgery is not a way to prevent the development of intra- and extracranial metastases but can be considered as an option for treating recurrences after surgery and radiotherapy. Radiosurgery using the Gamma Knife was associated with an earlier development of the second recurrence compared with strategies based on surgical treatment (*P* < 0.05). In this regard, surgical resection of recurrent hemangiopericytomas followed by external beam radiotherapy should be the first therapeutic option in these patients [29].

## Conclusions

We present an extremely rare case of a giant hemangiopericytoma/SFT in the area of the skull base (clivus)/craniovertebral junction and the results of its surgical resection using the transnasal transclival approach, which, in our opinion, was the method of choice. Immunohistochemical analysis of excised tumor tissue plays a key role in definitive diagnosis. Based on the histopathological findings, hemangiopericytomas can have three degrees of malignancy, namely, low, intermediate, and high risk of associated metastasis. The most effective treatment for hemangiopericytomas is the maximum possible resection followed by irradiation of the tumor bed with a TRD of at least 50 Gy. Considering the nonspecific and often ambiguous clinical manifestations, as well as the tendency for recurrence and metastatic spread of the tumor, patients diagnosed with a hemangiopericytoma require careful and long-term monitoring by a neurosurgeon and an oncologist.
